# A study on the clinical application of a patented perfusion and suctioning platform and ureteral access sheath in the treatment of large ureteral stones below L4 level

**DOI:** 10.1007/s11255-018-2049-9

**Published:** 2018-12-07

**Authors:** Chuance Du, Leming Song, Xiaoyuan Wu, Xiaolin Deng, Zhongsheng Yang, Xianxin Zhu, Lunfeng Zhu, Junrong He

**Affiliations:** 1 Department of Urology, The First Affiliated Hospital of Nanchang University, 17 Yongwaizheng Street, Nanchang, 330006 Jiangxi P.R. China; 2 Department of Urology, The Affiliated Ganzhou Hospital of Nanchang University, Ganzhou, 341000 Jiangxi China

**Keywords:** Intra-luminal pressure monitoring, Suctioning, Ureteroscopic lithotripsy, Large ureteral stones

## Abstract

**Purpose:**

The purpose of the study was to evaluate the efficacy and safety of a patented perfusion and suctioning platform and ureteral access sheath in the treatment of large ureteral stones (≥ 1.5 cm) below L4 level.

**Methods:**

We recruited 122 patients with large ureteral stones below L4 level at our hospital from December 2014 to June 2017. The patients were randomly divided into the study and control groups. Multiple operative and perioperative parameters were compared between the two groups.

**Results:**

The study group had shorter operation time, less cases of postoperative fever, lower serum levels of PCT, IL-6 and BET within 24 h after surgery, as well as less number of cases receiving secondary surgery than the control group. Moreover, the former had a significantly higher stone clearance rate than the latter (*P* < 0.05; *t*-test or *χ*^2^ test).

**Conclusions:**

The patented perfusion and suctioning platform and ureteral access sheath are safe and effective in treating large ureteral stones (≥ 1.5 cm) below L4 level.

## Introduction

Transureteral holmium laser lithotripsy is considered as a safe and effective technology for treating ureteral stones with small invasiveness and fast patient recovery. But for some cases with large ureteral stones, they are complicated by high stone burden and urothelial hyperplasia, which may result in prolonged operation time, ureteral injury, surgical failure, postoperative fever, high residual stone rate, and long stone expulsion time [1]. Furthermore, an increase in intrarenal pressure due to continuous perfusion may lead to postoperative fever, urinary tract infection or even severe sepsis [2]. As the stone fragments accumulate within the surgical field, it will cause disturbance to the surgery, leading to secondary injury, postoperative ureteral stricture and hydronephrosis [3, 4]. Measures to solve these problems include control of intrapelvic pressure, prevention of stone translocation, timely removal of the stone fragments, clearance of the surgical field and reduction of ureteral injury. Therefore, the treatment of large ureteral stones remains complicated and challenging for urological surgeons.

In order to improve the safety and efficiency for treating large ureteral stones, we have developed a patented perfusion and suctioning platform (Patent Number ZL201410041761.1) and ureteral access sheath (Patent Number ZL201420055134.9) for ureteroscopic lithotripsy (hereinafter referred to as the patented system). This patented system is used for monitoring intra-luminal pressure and ureteroscopic holmium laser lithotripsy under negative pressure suctioning by automatically maintaining the intrapelvic pressure at a low negative level. From December 2014 to June 2017, we carried out a prospective and randomized study to evaluate the safety and efficacy of the patented system in treating large ureteral stones below L4 level in China.

## Materials and methods

### Patients

The study was approved by the ethics committee of the Affiliated Ganzhou Hospital of Nanchang University (Project No. GZSRMYYL20140121). Informed consent was obtained from all subjects before surgery. We recruited 122 patients with large ureteral stones (diameter ≥ 1.5 cm) below L4 level at our hospital from December 2014 to June 2017. Before surgery, all patients underwent routine physical examinations, blood test, urine analysis, B-mode ultrasound of the urinary system, kidney, ureter and bladder (KUB) X-ray, intravenous urogram (IVU), and computed tomography (CT) scan of the urinary tract. The computed tomography (CT) value of stones was measured; the size and position of the stones were determined, and renal function and surgical contraindications were assessed. The patients were randomly divided into two groups. The study group (*n* = 62) received ureteroscopic holmium laser lithotripsy under negative pressure suctioning using the patented system; intra-luminal pressure was monitored during surgery. The control group (*n* = 60) received conventional transurethral ureteroscopic holmium laser lithotripsy. Semi rigid ureteroscope was used for the surgeries in the study and control groups. Preoperative serum levels of procalcitonin (PCT), interleukin-6 (IL-6), and endotoxin (BET) were measured.

### The patented perfusion and suctioning platform

**Fig. 1:**
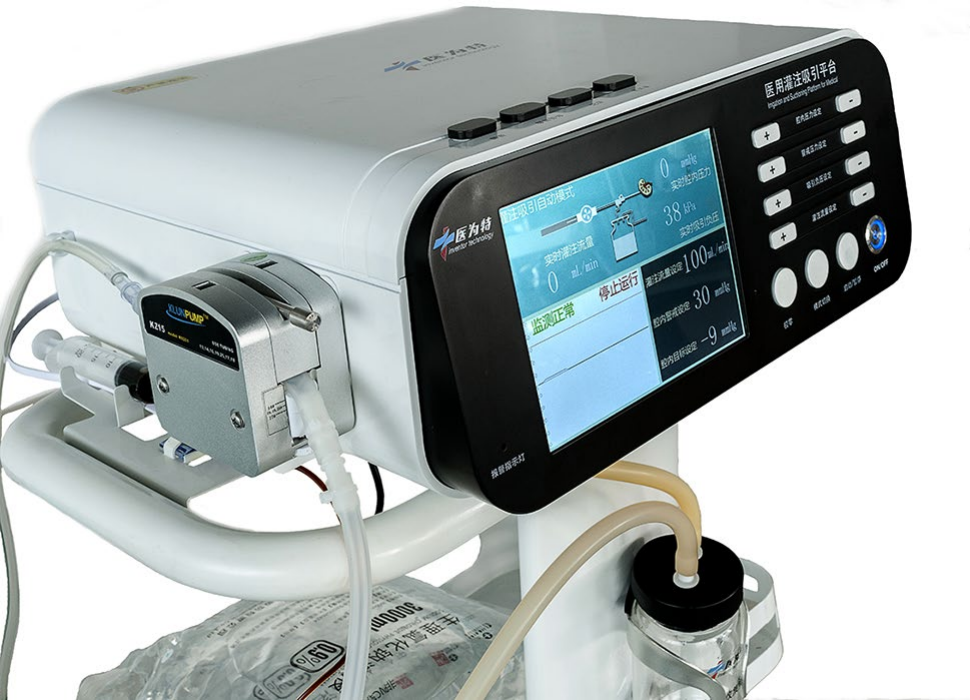
The patented perfusion and suctioning platform with pressure feedback and control function

**Fig. 2:**
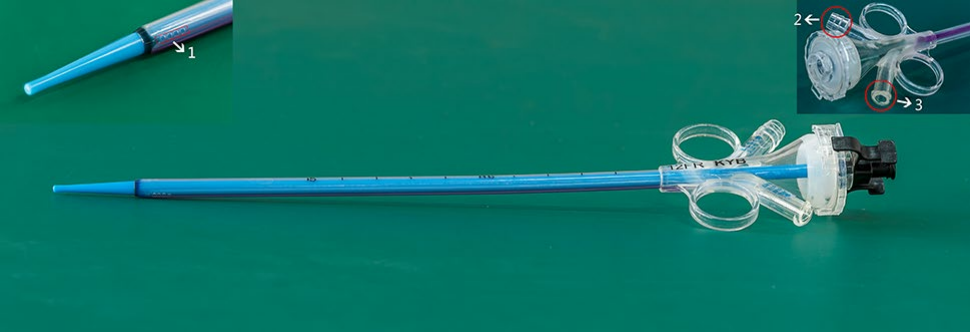
Structural diagram of the ureteral access sheath (1. Pressure detection and feedback channel at the front end of the sheath; 2. Negative-pressure suctioning channel; 3. Pressure-measuring interface)

The patented perfusion and suctioning platform was developed by Jiangxi Inventor Technology Co., Ltd. The perfusion and suctioning platform consists of a main control unit, perfusion device, suctioning device and pressure feedback device. The platform allows setting of perfusion flow rate, control pressure, alarming pressure and maximum pressure level (Fig. 1). The ureteral access sheath has an inner diameter equal to a F12 sheath and an outer diameter equal to a F14 sheath (Fig. 2). With a length of 30–45 cm, the suctioning sheath is installed with a pressure sensor at the front end and two connection channels at the back end, which are respectively connected to the pressure monitoring, feedback device and the negative-pressure suctioning device. The latter is responsible for sucking out the stone fragments during lithotripsy, and the former is for real-time monitoring, feedback and automatic adjustment of pressure in the operation area. The pressure values acquired by the pressure sensor on the suctioning sheath are fed back to the main control unit, which then adjusts the negative-pressure suctioning to maintain a safe intra-luminal pressure for the operation area. If the pressure of the operation area exceeds the alarming pressure level due to obstruction by the stone fragments or blood clots, the platform will give an alarm. If the pressure exceeds the specified maximum pressure level, the platform will shut down automatically to stop perfusion.

### Surgical procedures

#### Surgery for the study group

The study group received ureteroscopic holmium laser lithotripsy under negative pressure using the patented system. Intra-luminal pressure was monitored during surgery. The patients took a lithotomy position, and F7/8.4Storz ureteroscope was inserted into the ureter on the affected side until reaching the site of ureteral stones. The distance from the stones to the external ureteral orifice was measured and the zebra guidewire was inserted. Then, the ureteral access sheath was delivered along the zebra guidewire. In case of ureteral stricture, the ureteral access sheath was inserted after dilation to a depth equal to the distance from the stones to the external ureteral orifice. Next, the F7/8.4Storz ureteroscope was inserted, making sure that one end of the suctioning sheath had reached the stones. The pressure-measuring and suctioning interfaces of the suctioning sheath were connected to the patented perfusion and suctioning platform by pressure sensor and suctioning tube, respectively. The platform applied full-auto mode. Platform parameters were configured as follows: perfusion flow rate (50–100 ml/min), control pressure within the operation area (− 15 mmHg to − 5 mmHg), alarming pressure level within the operation area (10 mmHg), maximum pressure level (20 mmHg). F7/8.4Storz ureteroscope was inserted during the procedure. Lithotripsy was performed using lumenis 550 µm holmium laser fiber with a power of 0.6–0.8 J/ 25–30 Hz for in situ pulverization. Stone fragments smaller than the gap between the ureteroscope and suctioning sheath were automatically sucked out; those larger than the gap were gradually sucked out along with the withdrawal of the ureteroscope. After stone clearance, a 4.5–6 F double-J stent was inserted under the guidance of a guidewire.

#### Surgery for the control group

Patients in the control group received routine transurethral ureteroscopic holmium laser lithotripsy in a lithotomy position. F7/8.4Storz ureteroscope was inserted into bladder through the direct vision internal urethrotomy. The ureteral orifice on the affected side was located and the zebra guidewire was inserted into the ureter. Then, the ureteroscope was inserted under the guidance of zebra guidewire to locate the stones, so as to check whether there is ureteral stricture or hyperplasia. Lithotripsy was performed using lumenis 550 µm holmium laser fiber with a power of 0.6–0.8 J/25–30 Hz for sufficient pulverization. Some stones were removed with forceps or stone retrieval basket. Double J stent was inserted.

All surgeries were performed by the same surgeon. The operation time and the number of cases with ureteral perforation were recorded. Ureteral perforation was diagnosed if peri-ureteral fat was seen at the site of perforation. Routine blood test and measurements of PCT, IL-6, and BET were performed within 24 h after the surgery. The patients received plain abdominal X-ray 1 month after the surgery to assess residual stones. If no residual stones ≤ 4 mm were present, the patient was defined as stone-free. The double J stent was removed 2–4 weeks after surgery. All cases were followed up for 6 months after surgery to see if there is any ureteral stricture. For those with residual stones or ureteral stricture, extra-corporeal shock wave lithotripsy (ESWL) or secondary surgery was given. Those with residual stones received further observation.

### Statistical analyses

All statistical analyses were done using SPSS 18.0 software. Measurements were expressed as mean (standard deviation, SD). Student’s *t* test and *χ*^2^ test were used to test statistical difference among groups. *P* < 0.05 was taken to indicate significant difference.

## Results

Patients in the study group were aged 27–66 years old, with a mean age of 47.36 (SD, 13.16) years. The mean diameter of the stones was 21.88 (SD, 4.93) mm, 21 cases had stones in the upper ureteral segment (from L4 level to the upper margin of the sacroiliac joint), while 15 cases had stones in the middle ureteral segment and 26 cases in the lower ureteral segment. Patients in the control group were aged 29–63 years old, with a mean age of 46.95 years (SD, 15.72). The mean diameter of the stones was 21.37 (SD, 3.61) mm. There were 20 cases with stones in the upper ureteral segment (from L4 level to the upper margin of the sacroiliac joint), 13 cases in the middle ureteral segment, and 27 cases in the lower ureteral segment. The comparison of the age, gender, stone size, CT values, preoperative PCT, IL-6 and BET levels of patients was statistically non-significant between the two groups (*P* > 0.05, *t*-test or *χ*^2^ test, Table 1).

**Table 1:** Comparison of preoperative clinical data between the two groups

Clinical data	Study group (*n* = 62)	Control group (*n* = 60)	Statistics (*t*, or *χ*^2^ test)	*P* value
Male [*n*]	37	36	0.03	0.86
Female [*n*]	25	24		
Age (year)	47.36 (13.16)	46.95 (15.72)	2.75	0.83
Maximum stone diameter (mm)	21.88 (4.93)	21.37 (3.61)	1.98	0.89
Preoperative PCT (ng/ml)	0.058 (0.013)	0.052 (0.02)	0.98	0.83
Preoperative IL-6 (pg/ml)	5.6 (3.9)	5.7 (4.1)	0.10	0.92
Preoperative BET (EU/ml)	0.59 (0.36)	0.58 (0.37)	0.08	0.93
CT value of stone (Hu)	1022.8 (215.3)	984.5 (226.8)	0.67	0.55

All patients received surgeries as planned. None had ureteral perforation in the study group; one case of postoperative fever (T 38.2 °C) was found; None of the cases had residual stones 1 month after surgery or ureteral stricture during 6-month follow-up or received secondary surgery. Among 60 cases in the control group, 2 cases had intraoperative ureteral perforation; 7 cases had postoperative fever (*T* > 38.5 °C); 3 cases had ureteral stricture; 5 cases received secondary surgery; and 2 cases received ESWL. The incidences of postoperative fever and secondary surgery in the study group were significantly less than those in the control group (*P* < 0.05, *χ*^2^ test, Table 2). Cases in the study group showed significantly less operation time, higher stone clearance rate and lower serum levels of PCT, IL-6, and BET within 24 h after surgery than the conventional lithotripsy procedure (*P* < 0.05, *t*-test, Table 2). The cost of the patented platform and a ureteral access sheath are about $50 000 and $110, respectively. The patented perfusion and suctioning platform can be used repeatedly. There was no significant difference in hospitalization cost and hospital stay between the two groups (*P* > 0.05, *t*-test, Table 2). The above results indicated the patented perfusion and suctioning platform and ureteral access sheath outperformed the conventional lithotripsy procedure.

**Table 2:** Comparison of postoperative parameters between the two groups [Mean (SD)]

Clinical data	Study group (*n* = 62)	Control group (*n* = 60)	Statistics (*t*, or *χ*^2^ test)	*P* value
Operation time (min)	25.3 (5.6)	47.2 (9.8)	28.57	0.00
Number of cases with postoperative fever	1	7	5.030	0.03
Number of cases with ureteral perforation	0	2	2.752	0.14
PCT within 24 h after surgery (ng/ml)	0.341 (0.25)	3.354 (1.57)	32.02	0.00
IL-6 within 24 h after surgery (pg/ml)	6.2 (4.1)	9.1 (5.2)	12.46	0.00
BET within 24 h after surgery (EU/ml)	1.57 (0.53)	3.51 (2.68)	11.57	0.00
Stone clearance rate (%)	100	81.7	35.83	0.00
Number of cases with ureteral stricture	0	3	3.18	0.08
Number of cases receiving secondary surgery	0	7	7.67	0.01
Hospital stay (day)	4.41 (1.15)	4.50 (1.59)	0.14	0.89
Hospitalization cost ($)	3401.2 (625.7)	3219.6 (724.5)	0.24	0.73

## Discussion

**Fig. 3:**
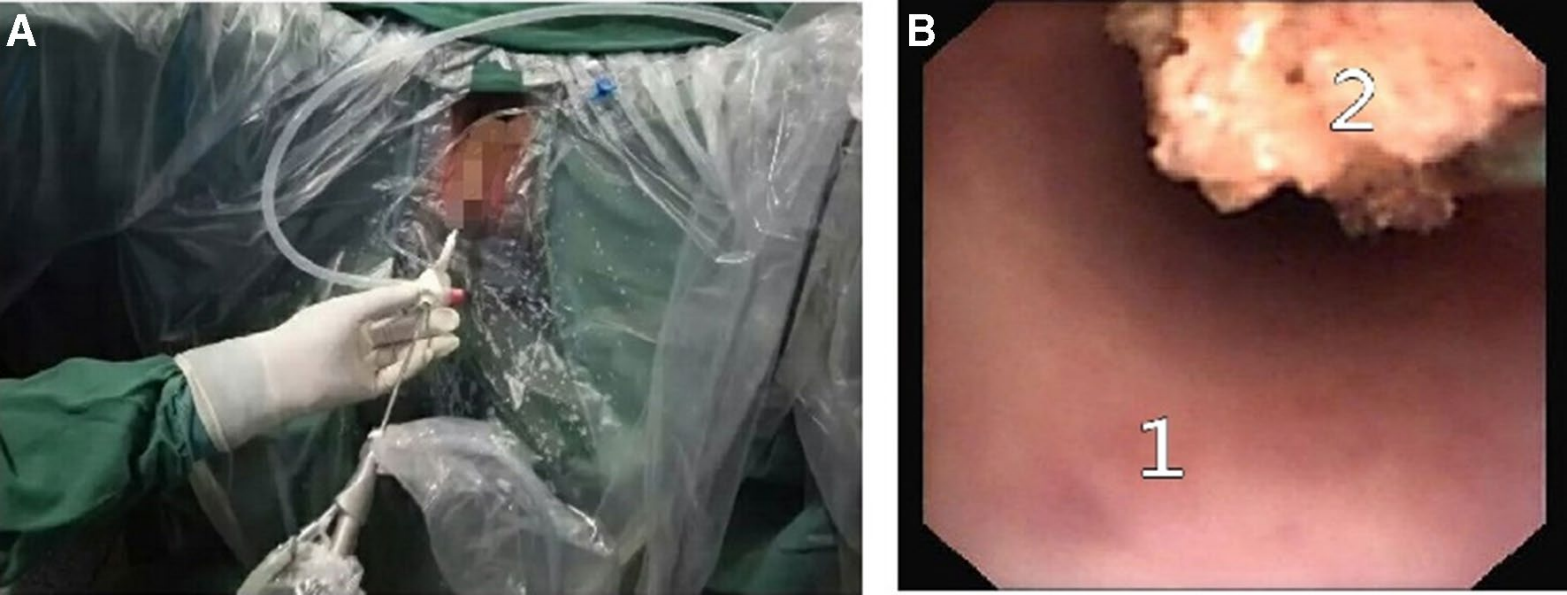
A surgery in the study group using the patented system. **a** Scene of surgery; **b** stones were being sucked out while withdrawing the ureteroscope (1. Ureteral access sheath; 2. Stone)

**Fig. 4:**
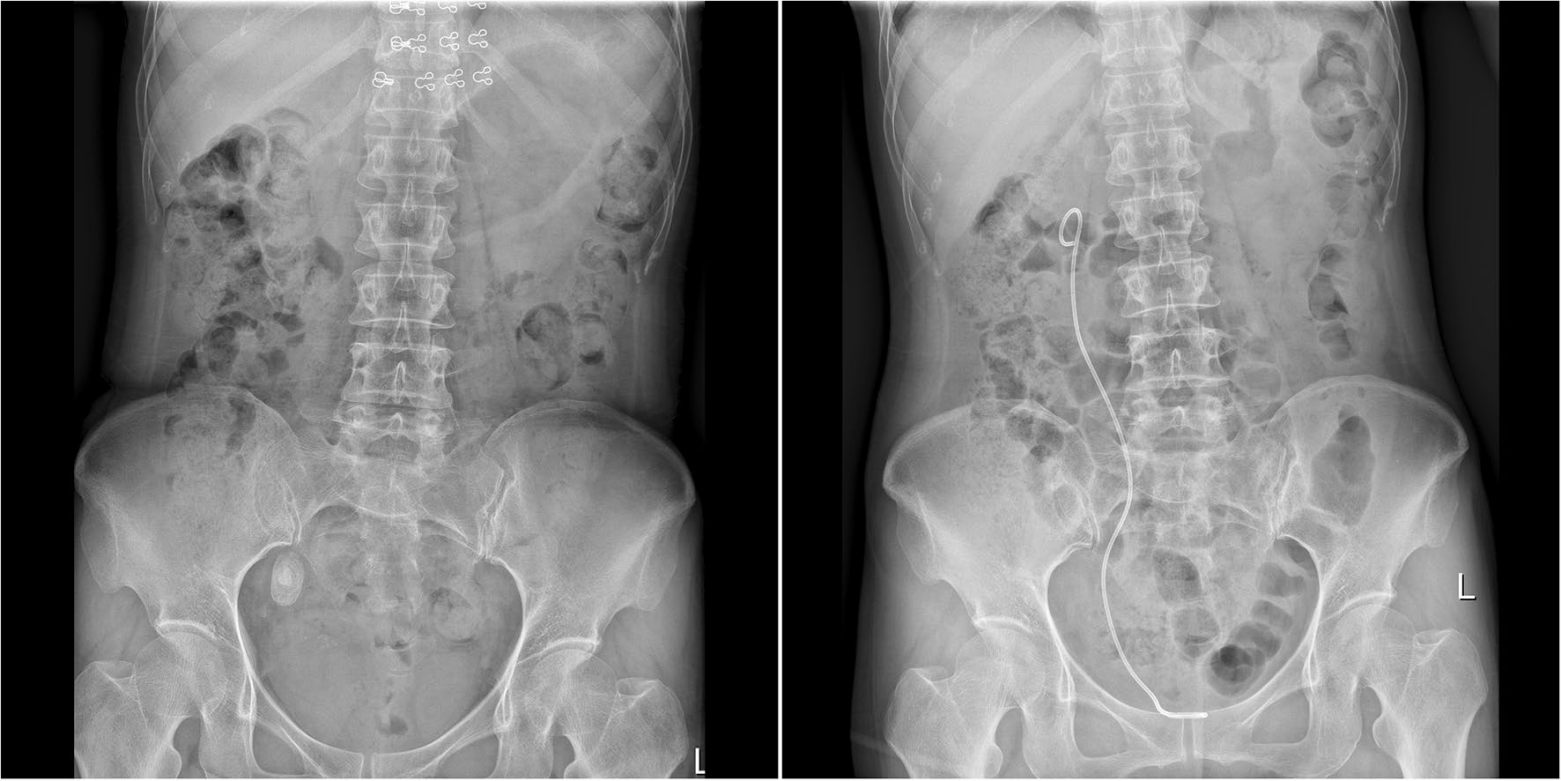
Preoperative and postoperative abdominal X-ray images in one case with large ureteral stones from the study group (left: before surgery; right: after surgery)

**Fig. 5:**
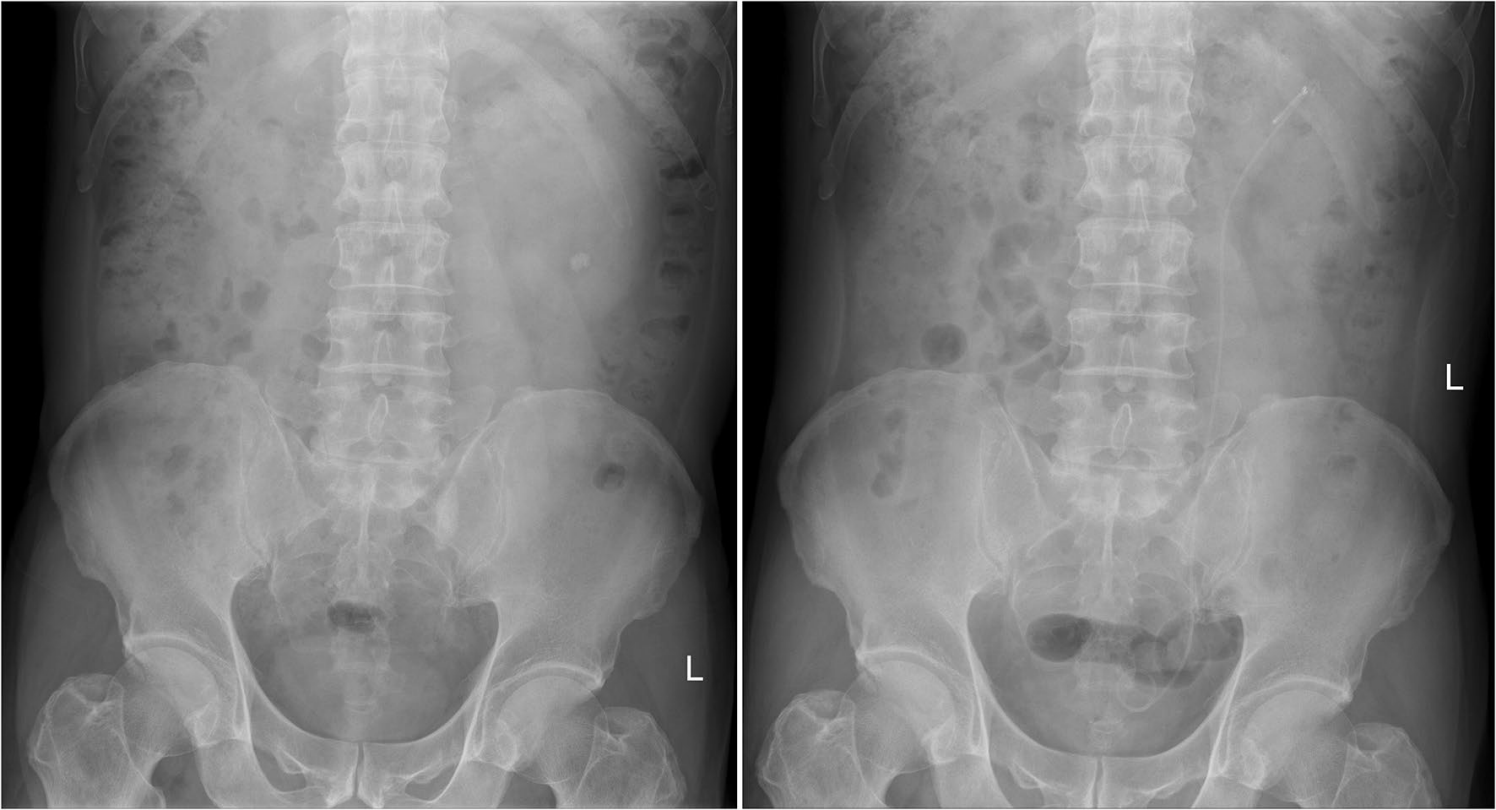
Preoperative and postoperative abdominal X-ray images in another case with large ureteral stones from the study group (left: before surgery; right: after surgery)

Urinary calculi (urinary stone) is one of the most common diseases in the department of urologic surgery. It is estimated that ureteral stone accounts for 12.3% of the cases with urinary stone diseases [5]. Large ureteral stones above the L4 level arep; one case of postoperative fev more properly removed by percutaneous nephrostolithotomy [6], while those below the L4 level can be removed by transureteral ureteroscopic procedure. However, large ureteral stones are usually associated with various problems. For example, there are too many stone fragments; the stones can be hardly discharged after surgery; secondary injury may be caused to ureters due to repetitive performance of ureteroscopic procedure for a thorough lithotripsy; the thermal effect associated with holmium laser lithotripsy may cause ureteral injury and postoperative ureteral stricture [7–9]; it may take longer time to discharge the stones after surgery due to large size of stone, and some may even need secondary surgery, and the success rate of the surgery remains low [10]. According to Gdor et al. study [11], the success rate of transureteral ureteroscopic holmium laser lithotripsy was only 56% (5/9) for large ureteral stones, and there was only one successful case in the proximal ureteral segment (1/3). Similarly, other scholars reported that for large proximal ureteral stones, the initial success rate of holmium laser lithotripsy was only 41.4% (12/29) [12]. To address these problems, we have developed the patented perfusion and suctioning platform with ureteral access sheath. Using this system, lithotripsy and suctioning are conducted simultaneously, and large stone fragments are sucked out when withdrawing the ureteroscope (Fig. 3). The stone fragments can be completely removed during surgery, and no residual stones are left (Figs. 4, 5).

It has been reported that 28%–60% of the ureteral stones occur in the upper ureteral segment [13, 14] and 3%–15% in the lower ureteral segment [15, 16]. In these positions, the stone fragments may be dislocated to the proximal end during ureteroscopic lithotripsy. This is especially true for large stones. Dislocation of ureteral stones will prolong operation time, reduce the stone clearance rate and increase medical cost [17, 18]. In our study, perfusion and suctioning were carried out simultaneously, large stone fragments are sucked out when withdrawing the ureteroscope. A continuous low negative pressure was maintained during the procedure via pressure control. Thus, a balance between perfusion and negative pressure suctioning was kept automatically, so that the upward movement of the stones was prevented and the lithotripsy was made easier. Cabrera et al. [19] believed that strategies to prevent dislocation of the ureteral stones must satisfy three requirements: effective in preventing dislocation of the stones; easy to operate; capable of resisting the impact of stone fragments without causing damage to the device. Our study has demonstrated that the patented system can meet the above requirements.

Infection is one of the most common complications associated with ureteroscopic holmium laser lithotripsy; in some severe cases, it may even cause life-threatening urine-derived sepsis. In our study, cases of the study group received monitoring of intra-luminal pressure during surgery, which automatically maintained continuous perfusion and suctioning while keeping a low intra-luminal negative pressure. Once the intra-luminal pressure increases, perfusion will be terminated automatically to prevent risk arising from high intrapelvic pressure, such as infection [20]. The larger the ureteral stones, the longer time the lithotripsy takes and the higher probability of systemic inflammatory response syndrome occurs [21, 22]. Serum PCT, IL-6, and BET are usually used as infectious indicators. We observed no apparent increase of these indicators in the study group. Moreover, the number of cases with postoperative fever was also significantly less in the study group compared with the control group. The advantages of the innovative lithotripsy procedure are prominent in the control of intra-luminal pressure, facilitating negative pressure suctioning and preventing infection.

The perfusion volume in the study group was higher than that in the conventional ureteroscopic holmium laser lithotripsy. By using the new system, thermal damage caused by holmium laser could be prevented. The stone fragments produced by lithotripsy were removed immediately by negative pressure suctioning, so that lithotripsy would not be disrupted by stone fragments or bleeding. Therefore, the surgical field was kept unobstructed and the surgical efficiency was promoted without causing ureteral perforation. In addition, the ureteral access sheath served as a stent, which prevented the invagination of the polyp below the stones after clearing the stones and hence avoided the disturbance to lithotripsy.

No significant difference was found in hospitalization cost and hospital stay between the study and control groups, suggesting application of the patented system doesn’t significantly increase economic burden of patients. The patented platform is intelligent and easy to operate; therefore, the main obstacles of popularizing this technology lie in the placement of ureteral sheath; the surgeons should have experienced skills in placing ureteral sheath. Therefore, the patented perfusion and suctioning platform and ureteral access sheath are safe and cost-effective in treating large ureteral stones (≥ 1.5 cm) below L4 level.

To conclude, we present a patented system to treat large ureteral stones (≥ 1.5 cm) below L4 level. The patented system shows several advantages in treating large ureteral stones (≥ 1.5 cm) below L4 level, including shorter operation time, lower incidences of postoperative fever and secondary surgery as well as higher stone clearance rate.
